# Modeling interpersonal perception in dyadic interactions: towards robot-assisted social mediation in the real world

**DOI:** 10.3389/frobt.2024.1410957

**Published:** 2024-11-28

**Authors:** Hifza Javed, Weinan Wang, Affan Bin Usman, Nawid Jamali

**Affiliations:** ^1^ Honda Research Institute USA, Inc., San Jose, CA, United States; ^2^ Department of Electrical and Computer Engineering, Rutgers University, Piscataway, NJ, United States

**Keywords:** relational affect, multimodal, multi-perspective, dyadic interaction, group dynamics, interpersonal dynamics, social mediation

## Abstract

Social mediator robots have shown potential in facilitating human interactions by improving communication, fostering relationships, providing support, and promoting inclusivity. However, for these robots to effectively shape human interactions, they must understand the intricacies of interpersonal dynamics. This necessitates models of human understanding that capture interpersonal states and the relational affect arising from interactions. Traditional affect recognition methods, primarily focus on individual affect, and may fall short in capturing interpersonal dynamics crucial for social mediation. To address this gap, we propose a multimodal, multi-perspective model of relational affect, utilizing a conversational dataset collected in uncontrolled settings. Our model extracts features from audiovisual data to capture affective behaviors indicative of relational affect. By considering the interpersonal perspectives of both interactants, our model predicts relational affect, enabling real-time understanding of evolving interpersonal dynamics. We discuss our model’s utility for social mediation applications and compare it with existing approaches, highlighting its advantages for real-world applicability. Despite the complexity of human interactions and subjective nature of affect ratings, our model demonstrates early capabilities to enable proactive intervention in negative interactions, enhancing neutral exchanges, and respecting positive dialogues. We discuss implications for real-world deployment and highlight the limitations of current work. Our work represents a step towards developing computational models of relational affect tailored for real-world social mediation, offering insights into effective mediation strategies for social mediator robots.

## 1 Introduction

Social mediator robots can play a pivotal role in shaping human interactions. They can facilitate human interactions by improving communication, building relationships, providing support, and promoting inclusion. To achieve these goals, the robot must produce behaviors that impact how humans interact with one another. However, for the behaviors to be effective in shaping human interactions, these must be in line with the interpersonal dynamics within these interactions. Therefore, it is crucial that the robot be able to “understand” humans and their interpersonal relationships with one another. This objective requires models of human understanding that consider the interpersonal states of the interactants and represent the interpersonal dynamics that exist between them.

Interpersonal dynamics may be captured by relational affect–a dyadic construct that represents affective states that an individual experiences from their interactions with others. According to Slaby (Slaby, 2019), relational affect “does not refer to individual feeling states but to affective interactions in relational scenes, either between two or more interactants, or between an agent and aspects of [her or his] environment.” Relational affect is a consequence of the interaction itself in the form of an interplay of gaze, gesture, tone of voice, etc. (Slaby, 2019), and it focuses on the observable expressions of affect between the interactants rather than the individual internal experiences of emotion. This shift from viewing affective behaviors as reflections of internal states to observable expressions of interpersonal states enables the design of studies that deliberately target the capture of interpersonal affective states that represent the underlying dynamics in human-human interactions.

Although advances in the field of Affective Computing have spurred numerous investigations into human affect recognition, it is important to note that traditional methods for affect detection in individuals (Calvo and D’Mello, 2010) may not appropriately capture interpersonal dynamics (Jung, 2017) that are crucial for social mediation applications. Some studies use a combination of individual-level affect states to estimate group-level affect (Veltmeijer et al., 2023). In doing so, however, only the *individual, internal* affect of each group member is represented. This is different from relational affect which is meant to capture *interpersonal* affective states. This gap is also reflected in the available affect recognition datasets, a majority of which are labeled for internal affective state of an individual rather than relational affect states resulting from interpersonal interactions. In addition to the limited ability to capture interpersonal dynamics, affect recognition datasets often have the disadvantage of being collected in controlled, laboratory settings that limits their applicability for affect-based social mediation applications in the real world. We discuss this in detail in Section 2.

For robot-assisted social mediation applications, an understanding of relational affect can be crucial. A model of relational affect can enable a real-time understanding of continuously evolving interpersonal dynamics within human interactions. This enables the detection of salient interpersonal events, informing the timely generation of meaningful, contextually-appropriate mediation actions from the robot. It also paves the path for real-world deployment of a social mediator robot, which can detect changes in relational affect and act autonomously in response to these changes to perform effectively in its mediation role.

**FIGURE 1:**
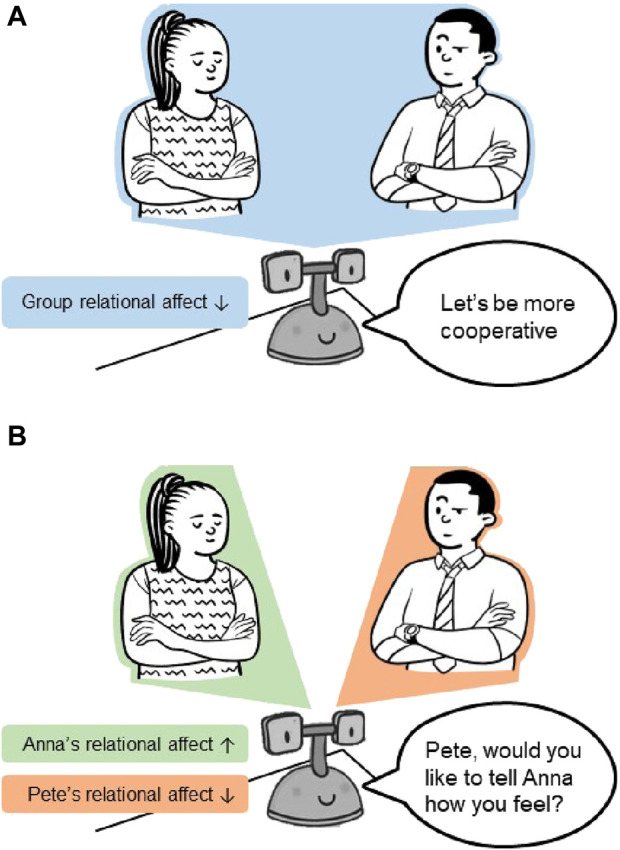
A comparison of potential mediation strategies resulting from a group-level view versus a multi-perspective view of relational affect within an interaction. **(A)** A group-level view of relational affect yielding generic, undirected mediation prompts from the robot. **(B)** A multi-perspective view of relational affect yielding directed, targeted mediation prompts from the robot.

Additionally, for social mediation to be effective, a mediator robot must be able to understand the interpersonal perception of each interactant towards the other. By capturing these individual perspectives, a nuanced view of the interpersonal dynamics becomes available, offering the robot crucial insights to inform its mediation strategy. In contrast, if only a group-level measure of overall relational affect is available, the robot may only be able to mediate with generic, undirected prompts (see Figure 1A). However, equipped with a multi-perspective view of relational affect, the robot can produce directed, targeted prompts that are sensitive to the intricacies of interpersonal interactions (see Figure 1B).

In this work, we develop a multimodal, multi-perspective model of relational affect that can be useful for robot-assisted social mediation in the real world. We utilize an audiovisual dataset^1^ collected using the COntinuous Retrospective Affect Evaluation (CORAE) tool from Sack et al. (2023), which contains video recordings of dyadic social interactions. For this work, the choice of dataset is indeed a critical one in order to ensure applicability of the developed relational affect model to real-world mediation scenarios. We discuss this dataset and its suitability for this work in Section 3.

In this paper, we utilize the video data from this dataset to extract relevant features to represent interpersonal dynamics in dyadic interactions. To model relational affect in dyadic interactions, we take a multimodal, multi-perspective approach that takes as input sequential audiovisual features to capture the interplay of affective behaviors that represent relational affect. We design our feature space to represent the interpersonal perspectives of both interactants to enable a model that can predict relational affect for each interactant. We then evaluate this model and assess its potential to inform social mediation applications in the real world. Motivated with the goal of deployment in real-world social mediation applications, this work takes the first steps towards developing a model of relational affect tailored for use in uncontrolled settings.

## 2 Related work

### 2.1 Traditional datasets for affect recognition

Facilitated by advances in the field of affective computing, a large body of research on affect prediction has emerged in recent years. As a result, a multitude of behavioral datasets collected for affect recognition purposes have become available that are labeled for internal affective states. In general, a fundamental assumption made during the collection and annotation of many of these datasets is that affective expressions are reliable indicators of an individual’s internal state. This assumption explains the popular annotation strategy that draws a one-to-one mapping between an emotional expression and the experienced internal emotion. However, it has been suggested that affective expressions alone may not be sufficient to reliably infer internal affective states (Fernandez-Dols et al., 1997).

In addition, affective expressions in these datasets are largely collected in controlled laboratory settings, either 1) performed by actors through exaggerated behavior (Lucey et al., 2010; Noroozi et al., 2017), or 2) spontaneously elicited through audiovisual stimulation (Zhalehpour et al., 2016; Busso et al., 2008). In both cases, these expressions may not resemble natural affective expressions that result from affective experiences in the real world. Moreover, relying on oversimplified representations of affect resulting from exaggerated performances can lead to an overly simplistic annotation process, which can also give a biased view of the performance of computational models trained on this data. Both approaches are likely to yield models that may not generalize to real-world applications.

Some datasets are collected to reflect uncontrolled, in-the-wild affective expressions by sourcing image data from the internet (Mollahosseini et al., 2017; Barsoum et al., 2016), the labels for the affective expressions are predominantly provided by annotators that are external to the affective experiences being labeled, which can introduce a disconnect between the label and the actual affective experience. Computational models trained on such data may show higher performance than on data from real world affective experiences since 1) the image data sourced from the internet may still be drawn from performed affective expressions, and 2) the labels, which often come from trained annotators who follow the same set of instructions. This leads to a high level of consistency in the labels applied to the expressions captured in the images. This consistency can lead to computational models that perform well on the test data. However, it may also limit their ability to generalize effectively in real-world scenarios.

Laboratory settings may result in higher data quality that may yield computational models that perform well. However, their applicability to affect recognition in the wild and subsequent social mediation applications is limited. In our study, we use a dataset in which the only instruction given to the participants was related to the interaction task. The participants were free to engage in the interaction in a natural setting from the environment of their choice.

### 2.2 Affect recognition in group settings

A small subset of the available affect recognition datasets enable an evaluation of group affective behavior. Braley and Murray (2018) collected the Group Affect and Performance (GAP) dataset from small group meetings and used human annotators to provide labels of satisfaction, group member influence, and binary sentiment based on utterance analysis. However, this dataset is developed primarily to study group performance rather than affect.

Mou et al. (2019b) use the multimodal AMIGOS dataset (Miranda-Correa et al., 2018), which was collected to study affect, mood, and personality in group settings. The authors studied how the affect of an individual may be influenced by their group members. However, this study was done in a laboratory setting within an audience context where all individuals watched movie clips together without interacting, as the participants’ physiological states are tracked with wearable sensors. The controlled environment, stimulation techniques, and the use of obtrusive sensors make this setting diverge from the uncontrolled experiences in the real world.

Additional datasets are available to study group affect, such as GAF (Ghosh et al., 2019) or VGAF (Sharma et al., 2019) that source data from the web to mimic uncontrolled, in-the-wild settings. Ghosh et al. (2020), Sharma et al. (2019), and Liu et al. (2020) used pretrained image classification models to estimate levels of group cohesion and emotion in the GAF and VGAF datasets. The labels in these datasets capture a single group-level measure of affect and do not represent each individual’s perspective of relational affect.

Few studies have explored affect to capture interpersonal dynamics in human-human interactions. Chen et al. (2022) collected the Dyadic Affect in Multimodal Interaction–Parent to Child (DAMI-P2C) dataset to study parent-child engagement during story reading activities. The parent-child interaction is centered around a tablet device with the reading activity, making behaviors such as gaze and speech task-specific and difficult to generalize to other social interactions. Semnani-Azad and Nouri (2015) collect a bespoke dataset containing dyadic interactions over a negotiation task. This is the only dataset we found that was labeled for relational rather than individual, internal affect. They employ an SVM classifier on nonverbal behavioral data such as facial features and body language to predict relational affect and degree of participant involvement while negotiating. However, the participants were given instructions to act in negative or positive relational affect states prior to the interaction, suggesting the possibility of captured behaviors deviating from real world affective behaviors.

These models of group affective behavior, their datasets and annotation approaches are summarized in Table 1.

**TABLE 1:** Summary of related studies of group affect in terms of their dataset, experiment settings, and annotations of affects.

Citation	Dataset	Data type	Task type	Annotator	Annotations
Sack et al. (2023)	CORAE	Audio + video from online meeting	Dyadic uncontrolled negotiation	Participants	Relational affect (affective ratings toward partner)
Chen et al. (2022)	DAMI-P2C	Audio + video + transcription from cameras	Parent-child guided story reading	External annotator	Individual affect (valence, arousal, engagement, coordination)
Ghosh et al. (2019)	GAF	Images from internet	Uncontrolled group activities	External annotator	Group affect (group emotion level)
Sharma et al. (2019)	VGAF	Audio + video from internet	Uncontrolled group activities	External annotator	Group affect (group emotion level)
Miranda-Correa et al. (2018)	AMIGOS	Audio + video + physiological signals from camera + neuro-physiological sensors	Grouped movie watching	Self-assessment + external annotator	Individual affect in group settings (audience context) (valence, arousal)
Braley and Murray (2018)	GAP	Audio + video + transcription from cameras	Grouped decision making	External annotator	Group affect (utterance-based sentiment), decision-making
Semnani-Azad and Nouri (2015)	(Unnamed)	Video from cameras	Dyadic controlled negotiation	External annotator	Relational affect (positive or negative)

### 2.3 Group dynamics in HRI

Several studies in Human-Robot Interaction (HRI) have investigated the use of a robot to influence the dynamics within a group interaction. Jung et al. (2015) studied how members of a group resolve conflict. They showed that when conflict arises due to personal violations, a robot functioning as an emotional regulator could assist in managing the conflict. Another study investigated conflict over object possession in child-play scenarios (Shen et al., 2018). However, these studies did not attempt to develop models of conflict resolution and instead relied on human experimenters to detect conflict and manually control the robot to produce relevant responses.

Tennent et al. (2019) used a microphone robot to mediate engagement in group interactions by rotating to face different participants upon detecting participation imbalance. The interactions were post-processed by human annotators that followed a coding scheme to label the dataset. Even though the robot behaved autonomously based on the detection of speech from participants, this work did not produce a computational model of group engagement.

Several studies from Gillet et al. (2021) investigate how group dynamics can be shaped by a robot. In one study, the authors evaluate the influence of adapted gaze behaviors on participation imbalance (Gillet et al., 2021) by measuring relative speaking times of the participants. In another study, the authors used a robot to facilitate inclusion within groups of children as they played a music-based puzzle game with online evaluation of collaboration (Gillet et al., 2020). However, these approaches do not explicitly measure interpersonal states (Gillet et al., 2021), with measures of dynamics that may be task-dependent, in this case, the puzzle game setup (Gillet et al., 2020).

While several other HRI studies have been designed to investigate and influence group dynamics, relational affect-based modeling of interpersonal dynamics is limited. By and large, the landscape of HRI research lacks studies that incorporate real-time assessment of interpersonal dynamics during group interactions, particularly in contexts where autonomous robot mediation is deployed in the wild. Our work attempts to address this gap by enabling real-time evaluations of interpersonal perception from the perspective of each interacting individual that can, in the future, facilitate autonomous mediation from a social robot in nonrestrictive, task-agnostic, natural environments.

## 3 Dataset overview

### 3.1 Interaction setting

**FIGURE 2:**
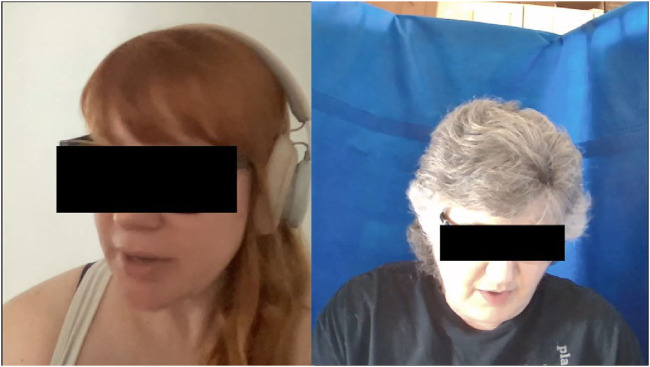
An example of the online dyadic interactions available in the dataset (Sack et al., 2023).

In this work, we utilize a dataset consisting of 30 dyadic interaction sessions (Sack et al., 2023). The study was conducted online through a video conferencing platform. An example of these interactions is shown in Figure 2. Upon joining the call, participants were presented with 13 possible reasons for why poverty exists, which cover several categories of explanations, including personal problems of poor people, lack of opportunities to escape poverty, exploitation of poor people, and bad fate (Shek, 2002). They were then asked to negotiate with their interaction partner to reach a consensus on their 5 most important reasons for poverty. They were given up to 10 min to come to an agreement. The participants were intentionally paired such that each person identified with a contrasting political ideology, creating an opportunity to study evolving interaction dynamics involving opinion-sharing, disagreements, persuasion, and empathy. Beyond imposing a time limit and providing the task instructions, the interactions were not structured or controlled. The goal was to use an uncontrolled setting to enable the development and evolution of natural conversational dynamics.

**FIGURE 3:**
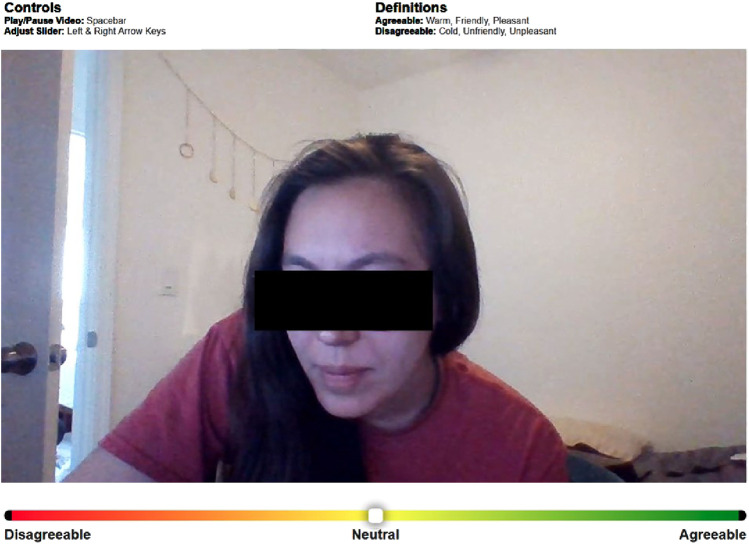
CORAE user interface showing the video replay and the slider interface designed to extract interpersonal ratings along a 15-point scale ranging from *disagreeable* (−7) to *agreeable* (+7).

At the end of the interaction, each participant was shown a video recording of their partner containing the audio from both participants. They were asked to retrospectively rate how their interaction partner came across to them through the course of the interaction. This rating serves as a measure of social distance between the interaction partners from each participant’s perspective. To this end, we used the CORAE tool (Sack et al., 2023), which is designed to extract intuitive and continuous ratings for interpersonal perception from the interactants themselves, with a slider bar interface representing a 15-point scale ranging from *disagreeable* (−7) to *agreeable* (+7) (Figure 3). The agreeableness ratings provided by the participants can be understood as an individual’s subjective interpretation of how their interaction partner came across to them during the interaction. Therefore, the rating serves as an indicator of how the relational affect manifested itself from the individual’s perspective and is used in this work as a measure for relational affect.

Details of the tool design and data collection process can be found here (Sack et al., 2023). The dataset was collected at Cornell University with IRB approval (IRB0143729). The authors obtained informed consent for collecting demographic and observational data from all study participants prior to the data release. However, as an additional precaution, we concealed the faces of participants in the figures to protect their identities.

### 3.2 Demographics

Of the 60 participants in the 30 dyadic interaction sessions, 28 were male, 31 female, and 1 non-binary. The ages ranged from 19 to 87 (M = 42.6, SD = 16.4). Race/ethnicity was primarily Caucasian/White (47), followed by Asian/Asian American (6), Hispanic/Latino (6), African/African American/Black (4), Middle Eastern/North African (1) and American Indian (1) (participants could select multiple options). Most participants were native English speakers (55), with 5 proficient users. For each online session, the dataset provided recordings of RGB videos and corresponding audio streams.

### 3.3 Dataset characteristics

This dataset offers a number of advantages over other available databases to study group social behaviors. Firstly, the dataset contains ratings from the participants themselves. This means that the provided labels may resemble the interpersonal experiences more closely than annotations from external observers who may offer an outsider’s view of the participants’ experiences. Consequently, this could facilitate a more accurate reflection of the interpersonal states. Secondly, the ratings in this dataset represent evaluations of the interaction partner’s behavior rather than one’s own, internal affective states. Thirdly, the dataset records interpersonal perception from the perspective of each individual, offering a view into each participant’s interpersonal perception. These ratings are continuous and provide a granular look into the interpersonal dynamics evolving during the interaction. This allows for a nuanced examination of the dynamics by enabling a study of the interplay between the affective behaviors exhibited by one participant and the corresponding responses from their partner, and how these unravel over time. Additionally, this provides a more fine-grained perspective compared to a single label to represent the interaction state as a whole, as is the case in some datasets mentioned in Section 2. These features make this dataset a suitable choice to develop models of relational affect for a social mediator robot that observes real-world social interactions and produces contextually-appropriate and impactful actions to successfully mediate.

On the other hand, the dataset also has limitations that pose some interesting challenges for developing computational models. Since the dataset was collected in uncontrolled settings, participants were allowed to connect using their personal devices from any environment that would not interfere with the conversation taking place. The hardware inconsistencies have implications for the feature extraction process, where the differences in quality of the recorded video and audio lead to inconsistent feature representations. Additionally, there are also variations in the camera angles and illumination conditions under which the videos were collected, potentially interfering with the subsequent visual feature extraction process. Another major challenge posed by this dataset is the large amount of subjectivity in both the affective expressions of the participants that may indicate interpersonal states and the retrospective ratings coming from the unique perspective of every participant. Lastly, the dataset consists largely of positively rated interactions compared to the negative. This imbalance causes difficulties in training machine learning or deep learning models, resulting in overfitting to the majority class. These challenges underscore the unique complexity of modeling human social states.

Given these dataset characteristics, Parreira et al. (2023) sought to understand the interpersonal trends captured in this dataset by performing a preliminary qualitative analysis on the correlations between ratings from the two participants in a session. They used cumulative ratings per participant in a time series to identify key interaction events, such as a sudden drop, opposing trends, and synchronicity to shed light on the contextual factors in the interaction that may explain the respective trends. Leveraging these findings, our current work focuses on developing a multi-perspective computational modeling approach to predict interpersonal affect states for social mediation applications.

## 4 Feature extraction and data pre-processing

In line with prior work that relies on audio and visual features to capture affect [see Poria et al. (2017) and Javed and Jamali (2023) for a review], our feature space is comprised of audiovisual features to capture a multimodal representation of relational affect in the dyadic interactions. A prior analysis of the interactions in this dataset found that verbal backchannels (“*mmhmm*,” “*okay*,” “*yeah*,” etc.) and non-verbal backchannels (*nodding*, *head tilt*, *eyebrow raise*, etc.) were important indicators of the interpersonal dynamics (Parreira et al., 2023). Based on these findings, we adapted our approach to feature extraction to emphasize the capture of short-term behavioral events occurring within the interactions. Each sample in the feature space includes audiovisual features from both participants to capture a joint representation of affective facial and/or verbal behaviors from the interaction partners. This joint representation is intended to reflect the interplay of salient affective cues exchanged between the two individuals during their interaction.

The inconsistencies in data collection environments and setups across the participants necessitated additional pre-processing steps to ensure data integrity. Since the two participants’ video and audio data were recorded disparately, further steps were implemented to align the extracted features to form a joint representation of participants’ features. The uncontrolled, online setup resulted in varying lighting conditions, camera angles, and face occlusions, as shown in Figure 5. These inconsistencies led to failures in extracting visual features, rendering some sessions unsuitable for analysis. Consequently, we utilized data from only 21 out of the 30 interaction sessions available in the dataset. This section discusses the details of the feature extraction and pre-processing implemented in our approach.

### 4.1 Visual features

**FIGURE 4:**
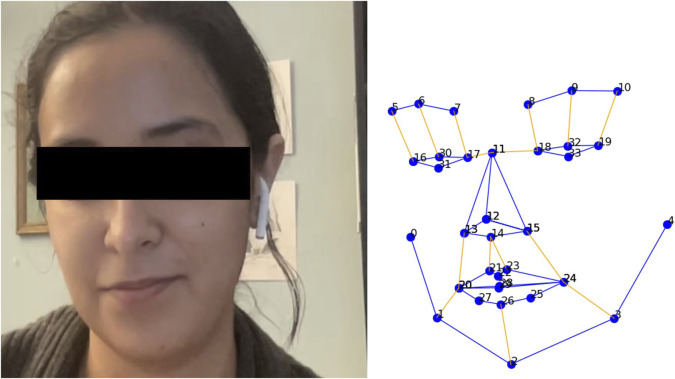
An example of the 34 2D facial landmarks extracted from a participant’s video data using iMotions.

Our visual features comprised of facial landmarks to represent key parts of a human face (nose, eyebrow, mouth, eyes, etc.). These were extracted using the facial expression analysis module from the iMotions^2^ software platform. The videos were processed at an average rate of 30 frames per second. This resulted in two-dimensional coordinates for 34 unique 2D facial landmarks, as depicted in Figure 4. This yielded 68 (×34, 34 years coordinates) features per participant, i.e., 136 visual features per data sample in the joint representation of both participants. A complete list of video features is provided as Supplementary Material.

### 4.2 Audio features

We extracted short-term acoustic features to capture the salient verbal expressions of relational affect, i.e., backchannels. Though both global and short-term acoustic features have been utilized in prior research, global-level acoustic features are limited in their ability to describe the short-term, dynamic variations (Busso et al., 2004) that commonly occur within human social interactions. Therefore, we extracted 34 audio features that represent low-level descriptors of voice, including Mel Frequency Cepstral Coefficients (MFCCs), energy, and spectral features. These features were extracted separately for each individual. A complete list of audio features is provided as Supplementary Material.

As in the case of video features, variations in the audio recording process, including differences in microphones or environmental conditions, can introduce inconsistencies that deteriorate the quality of the audio data available for feature extraction. Audio features were extracted at a sampling rate of 48,000 Hz and were then down-sampled to match the sampling rate of the video features to enable synchronization of the two modalities.

### 4.3 Data synchronization

**FIGURE 5:**
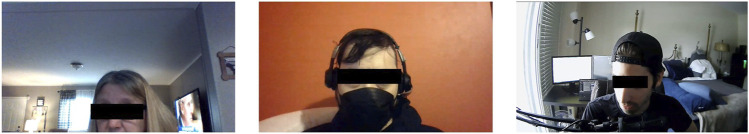
Some examples of the suboptimal conditions resulting from the uncontrolled data collection process that necessitate additional data pre-processing. **Left**: Suboptimal camera angle resulting in an incomplete view of the participant’s face, compounded by the occlusion from the glasses. **Center**: Occlusion from facial coverings. **Right**: Suboptimal lighting conditions causing uneven illumination, with one side of the face appearing significantly brighter than the other.

Due to the disparate recording of data for each participant within a session, we needed to align data samples to create a joint representation of audiovisual behaviors from both participants. In addition to the suboptimal conditions depicted in Figure 5, behaviors such as turning away from the camera or partially covering the face with a hand were observed frequently, resulting in facial occlusion. These instances presented challenges for the facial landmark extraction process, resulting in missing data in affected video segments. Consequently, only interaction segments where facial landmark data for both participants were available were included in our extracted dataset. The audio feature extraction process did not pose such difficulties. The remaining data samples were then synchronized through timestamp matching to yield the aligned dyadic interaction dataset.

### 4.4 Multi-perspective representation

**FIGURE 6:**
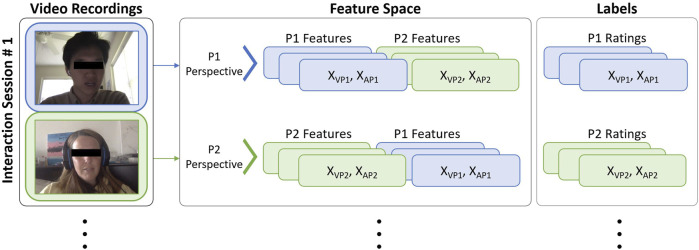
Structure of our feature space and labels that is used to capture multi-perspective representations of relational affect in dyadic interactions between P1 and P2.

In our study, we aimed to incorporate both participants’ perspectives on interpersonal affective states by leveraging the two sets of relational affect ratings. To achieve this, we adopted a method of alternating the order in which the features from each interactant were represented in the dataset. Specifically, we structured the dataset such that each session yielded two sets of sequences. In the first sequence, samples contained 68 landmarks $\left(X_{\mathrm{V P1}}\right)$ and 34 audio features $\left(X_{\mathrm{AP1}}\right)$ from P1 followed by 68 landmarks $\left(X_{\mathrm{V P2}}\right)$ and 34 audio features $\left(X_{\mathrm{AP2}}\right)$ from P2 and labeled with the rating from P1 $\left(Y_{P1}\right)$ . The second sequence contained 68 landmarks $\left(X_{\mathrm{V P2}}\right)$ and 34 audio features $\left(X_{\mathrm{AP2}}\right)$ from P2 followed by 68 landmarks $\left(X_{\mathrm{V P1}}\right)$ and 34 audio features $\left(X_{\mathrm{AP1}}\right)$ from P1 and labeled with the rating from P2 $\left(Y_{P2}\right)$ . This approach ensured that each interaction session was represented in the dataset from the viewpoints of both interactants. The resulting dataset structure is illustrated in Figure 6.

### 4.5 Additional pre-processing steps

In the extracted dataset, we noticed that, on average, there were slower changes in ratings recorded at the start and the end of the interaction session. This observation can be explained as follows: at the start, the slider is initialized at *neutral* (see Figure 3) and results in extended periods of neutral ratings as participants acclimate to the interface, and towards the end this behavior may be explained by possible fatigue experienced by the participants from providing the retrospective ratings. We, therefore, compute the average number of samples at the start (M_*start*_ = 214) and end (M_*end*_ = 975) of each session’s data before the first and after the last rating change occurs respectively and trim the data per session by eliminating these samples.

Lastly, we also perform feature normalization for each participant’s data. While normalizing the facial landmarks, we wanted to preserve the relationships between the 34 landmark coordinates and reduce between-subject differences. We opted for a technique that normalizes the landmark features by computing their relative distance from the nose and dividing them by the distance between the left and right jaw landmarks, to acquire a standardized landmark representation for modeling across subjects with different face sizes. The feature space consists of 204 features comprising of 68 facial landmarks and 34 audio features per participant.

## 5 Estimating relational affect

### 5.1 Data imbalance

**FIGURE 7:**
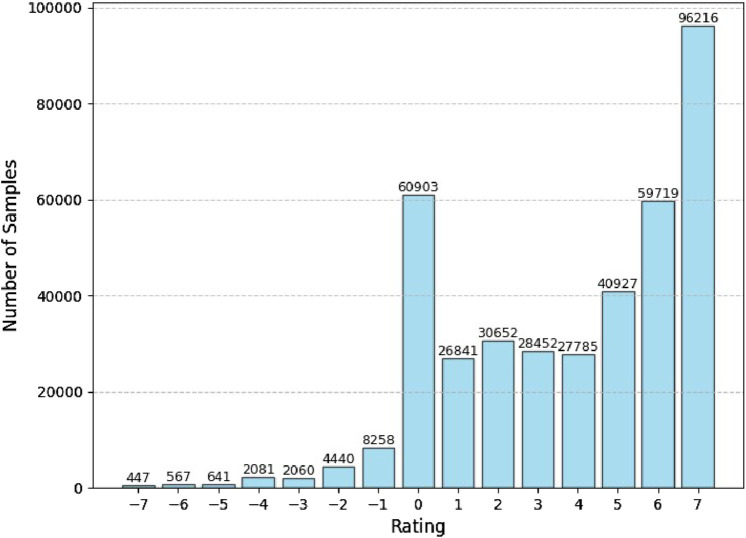
A barplot showing large imbalance in the rating classes available in our dataset, with a majority of positive ratings and a small minority of negative ratings.

In the dataset resulting from the techniques described in Section 4, we inspected the class distribution and found that our dataset contained a majority of positive ratings (79.6%), followed by neutral ratings (15.6%), and a very small minority of negative ratings (4.7%), as shown in Figure 7. Possible explanations for this observation are discussed in Section 3.3 Such a stark imbalance is known to cause difficulties with model training, making it prone to overfitting and leading to poor generalization. Therefore, addressing this class imbalance is critical for our model’s ability to produce reliable predictions of relational affect and facilitate effective social mediation.

We implemented a *weighted sampling* technique that is commonly used in machine learning to address class imbalance within datasets. Each sample is assigned a weight that is inversely proportional to the number of samples available in the dataset for its corresponding class. This increases the probability of the minority classes being sampled during training, effectively reducing the imbalance in the data.

Additionally, we also implemented a *weighted loss* function that weights the loss computed for different samples differently based on whether they belong to the majority or the minority classes. By assigning higher weights to minority classes than to majority classes, our weighted cross-entropy loss function penalizes the misclassification of negative ratings more than positive ratings. This increases the cost of misclassifying the negatively-rated samples, making the model more sensitive to these classes.

### 5.2 Relational affect zones for social mediation applications

The dataset collected by Sack et al. (2023) captures the interpersonal behaviors within dyads as interaction dynamics develop and evolve over time. The 15 rating classes provide a granular view into the exchange of affective behaviors and their correlation with interpersonal perceptions. While this offers valuable nuance in the study of group dynamics, the foremost prerequisite for a social mediation application remains the ability to detect the *need* for mediation. The need to mediate is correlated with the ability to detect the valence of the relational affect state. In its simplified form, this implies the ability to distinguish *low* relational affect from *moderate* and *high* relational affect. Since interactions in *high* or even *moderate* affective states indicate functional interaction dynamics that may not necessitate immediate intervention, it is most critical for mediation to be initiated if interpersonal perceptions are *low*. Motivated by this understanding, we decided to reduce the number of rating classes in our dataset, remapping them to three relational affect zones: Zone 1 (*low*), Zone 2 (*moderate*), and Zone 3 (*high*). This is in line with prior research from Section 2 that typically predicts 3 affective states.

Therefore, Zone 1 requires mediation with high priority since it is critical for the robot to take action when relational affect in an interaction is low. Zone 2 requires mediation with moderate priority since this state does not present a critical need for mediation. Zone 3 requires mediation with a low priority since the relational affect is high and represents well-functioning interpersonal dynamics that do not necessitate mediation from a robot.

**FIGURE 8:**
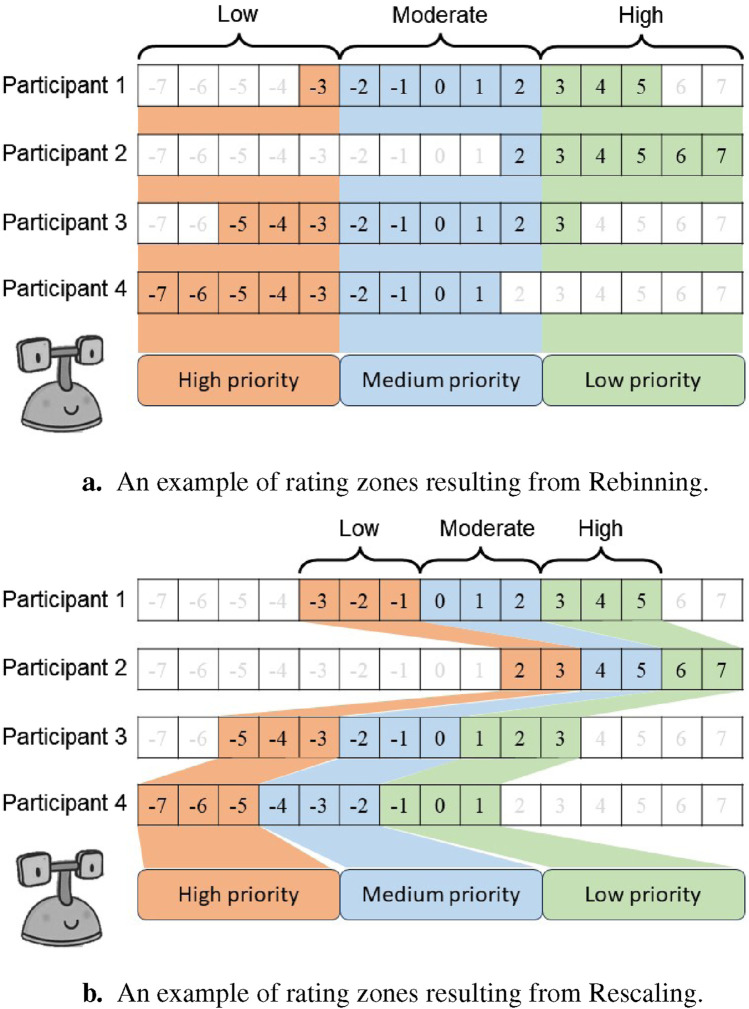
An example comparing the rating zones resulting from the Rebinning and Rescaling methods. Rebinning always produces three standard zones for all participants, as illustrated by the consistent boundaries in **(A)**. In contrast, the zones may vary between participants when Rescaling is used, as illustrated by the inconsistent zone boundaries in **(B)**. Zone 1 requires mediation with a high priority, followed by Zone 2 with moderate priority, and Zone 3 with low priority. **(A)** An example of rating zones resulting from Rebinning. **(B)** An example of rating zones resulting from Rescaling.

We implemented the following two techniques to remap the 15 original rating classes to the 3 zones that are useful for social mediation applications:

• **Rebinning**: This presents a naive approach to remapping classes in this dataset—it takes an objective view of the rating data and assumes that all participants use the scale the same way. This method takes the range of ratings provided by a given participant and divides it into three equal segments, as shown in Figure 8A. For example, consider a scenario where, out of the 15 possible rating options from −7 to +7 , Participant 1 provided ratings in the range of −3 to +5 , and Participant 2 provided ratings in the range of +2 to +7 . This technique always utilizes the full range of the scale to create the three standardized relational affect zones: *low* (red), *moderate* (blue), and *high* (green). In this case, the zone boundaries are always represented by integer values (*low*: −7 to −3 , *moderate*: −2 to +2 , *high*: +3 to +7 ) and are consistent between all participants irrespective of the range of rating scale they utilize. This implies that, for example, if a participant did not provide any negative ratings, the *low* category will contain zero samples.

• **Rescaling**: In contrast to Rebinning, this method takes a subjective view of the data by acknowledging that different participants may use the scale in different ways. Instead of using the full range of the scale, this method only takes the range of ratings provided by a given participant and divides it into three equal segments, as shown in Figure 8B. For example, consider the same scenario as above where, out of the 15 possible rating options from −7 to +7 , Participant 1 provided ratings in the range of −3 to +5 , and Participant 2 provided ratings in the range of +2 to +7 . The Rescaling technique divides this range into 3 zones: *low* (red), *moderate* (blue), and *high* (green), with the raw rating values within each zone varying between participants depending on the range of the scale they utilized. As such, the zone boundaries may not always be represented by integer values.

**FIGURE 9:**
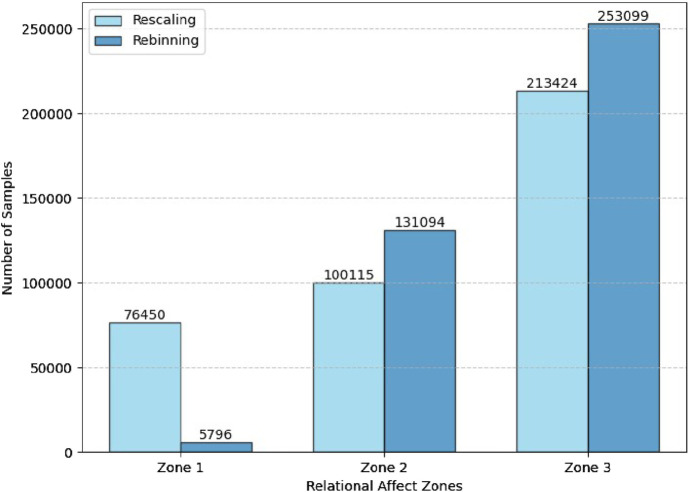
A comparison of the class distributions after implementing the rescaling and rebinning techniques.

The difference in definitions of zone boundaries and the correlation between the three relations affect zones with an action initiative priority from the mediating robot is evident in Figure 8. Additionally, the number of samples allocated to each rating zone after implementing the Rescaling and Rebinning methods are illustrated in Figure 9. As expected, the Rescaling strategy is better at balancing the class distribution compared to Rebinning. This is evident upon comparing the number of samples in Zone 1 resulting from the Rescaling (76,450) and Rebinning (5796). Therefore, we settled on using the Rescaling method to reduce the number of classes before proceeding to train our model.

### 5.3 Modeling

The relational affect ratings provided by the participants are closely tied to their observations of their partners’ behavior, including facial expressions, head movements, speech patterns, and nonverbal signals. This process is bidirectional, such that the participants’ own behavioral expressions are observed by their partners, influencing their affective evaluations. Thus, a dynamic feedback loop exists, shaping how relational affect is perceived in human relationships.

To capture affect-related information from sequential interactions between subjects, we employed an LSTM (Long Short-Term Memory) model. We chose a recurrent neural network architecture to capture the temporal occurrence of salient affective behaviors in the form of facial landmarks and audio features. The last hidden state of the LSTM, which represents high-level information aggregated from the input time window, was utilized together with the last fully connected layer to classify the relational affect state of each participant through the course of the interaction. We fine-tuned the LSTM model by conducting an extensive parameter search, ultimately setting the number of layers to 3 and the number of hidden neurons to 32. Additionally, a dropout rate of 0.1 was applied to the first two layers. We used a 5-s sliding window, each containing 150 samples, with a step size of 1 sample. Therefore, each input sequence consisted of 150 frames of facial landmarks and audio features from both participants in a session (204 features in total), corresponding to approximately 5 s of interaction. This allowed us to capture and model the participants’ responses to affective stimuli. The choice of brief 5-s windows is meant to capture important short-term interpersonal behaviors, such as backchanneling, and is in line with previous studies (Chen et al., 2022; Sharma et al., 2019).

The 3-layer LSTM model was trained using the Adam optimizer, with a learning rate of 1e−3 , a weight decay of 1e−4 , and a batch size of 64 to prevent overfitting. The model was trained for 500 epochs on the training set. To obtain a model that is sensitive to minority classes in an imbalanced dataset, we used the mean of class-specific recall as the performance metric for model selection. This is done to ensure the trained model is suitable for supporting mediation, particularly in *low* relational affect states (Zone 1), given that the test data is heavily biased toward *moderate* (Zone 2) and *high* (Zone 3) relational affect.

We use data from 18 sessions (36 unique participants) for training and 3 sessions (6 unique participants) for testing our model. This results in a 80/20 train-test split. It is important to note that the model is tested on unseen data, where the 3 test sessions are not represented in our training model. This is to evaluate the generalizability of the trained model to ensure applicability to real-world mediation scenarios involving humans that may not be a part of the current dataset.

**FIGURE 10:**
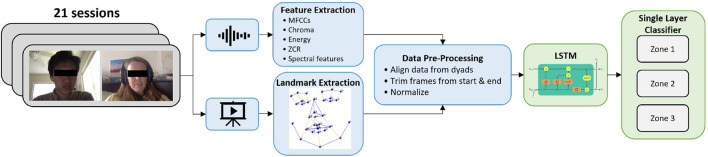
An illustration of our pipeline, including multimodal feature extraction, pre-processing, LSTM model, and predicted relational affect zone.

The complete pipeline for our modeling approach is shown in Figure 10.

## 6 Results

We trained our 3-layer LSTM network on 18 dyadic interaction sessions from the training dataset. On this training dataset, the model achieved an average recall of 90.17% and an accuracy of 89.05%. The results presented in the rest of this section are based on novel, unseen data from three hold-out sessions, consisting of behavioral data from six unique individuals. These results are a reflection of the model’s ability to learn from the behavioral expressions of relational affect from some individuals and use these to predict interpersonal states of unseen individuals.

Our model classifies the 3 relational affect zones at a class-specific recall of 45.99% and an accuracy of 40.49% . Figure 12 shows the class-normalized confusion matrix resulting from the evaluation of our model, which helps to visualize the recall and misclassifications of the three relational affect zones [see Simske (2019) and Mohamed et al. (2023) for class-normalized confusion matrices]. Majority of Zone 1 (41%) and Zone 3 (72%) samples were classified accurately, showing that our model is sensitive to *low* and *high* relational affect. A majority (58%) of Zone 2 samples were misclassified as Zone 3, showing that our model is frequently unable to distinguish *moderate* relational affect from *high*. Table 2 summarizes the performance of our model and compares it to other models of group affect or relational affect (discussed in Section 2). We explain our results in light of these models in Section 7. Overall, our findings underscore the unique complexity of modeling human interpersonal states in social interactions.

**TABLE 2:** Comparison between this study and related prior work on group affect or relational affect.

Research study	Dataset	Modalities	Task	Labeling type	Model	Affect categories	Performance
Ours	Dyadic interactions/w CORAE	Audiovisual features	Unstructured, uncontrolled social interaction	Self-provided ratings, continuous labels	LSTM	3 relational affect zones	Av. Recall = 45.99% Acc = 40.49%
Ghosh et al. (2020)	GAF3	Visual features	Sourced from the internet	Observer-rated, static labels	Inception-V3	3 group emotions	Acc = 85.58%
Sharma et al. (2019)	VGAF	Audiovisual features	Sourced from the internet	Observer-rated, static labels	Inception-V3 +LSTM	3 group emotions	Acc = 47.50%
Mou et al. (2019a)	AMIGOS	Visual features	Movie stimuli	Observer-rated, every 20s	LSTM	Binary arousal Binary valence	F1 = 0.71 F1 = 0.79
Semnani-Azad and Nouri (2015)	Unnamed dataset	Visual features	Controlled interactions/w assigned roles (act out affective states)	Observer-rated, static labels	SVM	Binary relational affec	Acc = 65%

Since the networks reported in this table were trained and evaluated on different datasets, direct comparisons are difficult. To address this, we trained and evaluated the Inception-V3 [used in Ghosh et al. (2020)] and Inception-V3 + LSTM [used in Sharma et al. (2019)] networks on our dataset. We used the same training and test sets as in our work, and used the same audiovisual feature space to produce predictions for the 3 rating zones. Inception-V3 achieved an average recall of 33.33% and an accuracy of 23.67%, and Inception-V3 + LSTM achieved an average recall of 33.33% and an accuracy of 23.84%. In comparison, our model (average recall of 45.99% and accuracy of 40.49%) outperforms these networks, highlighting the highly complex and nuanced interpersonal dynamics captured in our dyadic interaction dataset.

## 7 Discussion

We start with a comparison of our model with other comparable approaches as in Table 2. Firstly, Ghosh et al. (2020), Sharma et al. (2019), and Mou et al. (2019a), produce group affect states that are based on estimates of individual, internal affect, and do not consider relational affect to investigate group dynamics. Although Ghosh et al. (2020) achieve a significantly higher performance ( 85% accuracy), it is unclear if this performance comes from an evaluation on novel image data. Additionally, the 47% accuracy reported by Sharma et al. may be attributed to the fact that the labels are obtained from trained annotators who follow the same set of instructions to annotate affective states. This may present a skewed evaluation of model’s ability to predict affect and masks its true generalization capabilities. In contrast, our approach defines separate train and test sets, ensuring that our model is evaluated solely on novel data from previously unseen subjects. Generalizing to new data is a more complex challenge, and our methodology provides a more accurate assessment of our model’s ability to generalize its learning. Secondly, results from Mou et al. (2019a) are generated from experiments that investigate how individual affective states may be influenced by a group in an audience context that do not contain any interpersonal interactions between the subjects. In addition, they output binary affect levels compared to our 3-class output. Thirdly, Semnani-Azad and Nouri (2015) produce relational affect estimations on a dataset containing interactions performed in two different relational affect states. Since the subjects have assigned roles, it is possible that their behavior is exaggerated or influenced by external factors, potentially limiting the dataset’s representativeness of real-world scenarios. Additionally, the authors’ binary classifications contrast with our more nuanced 3-class output.

**FIGURE 11:**
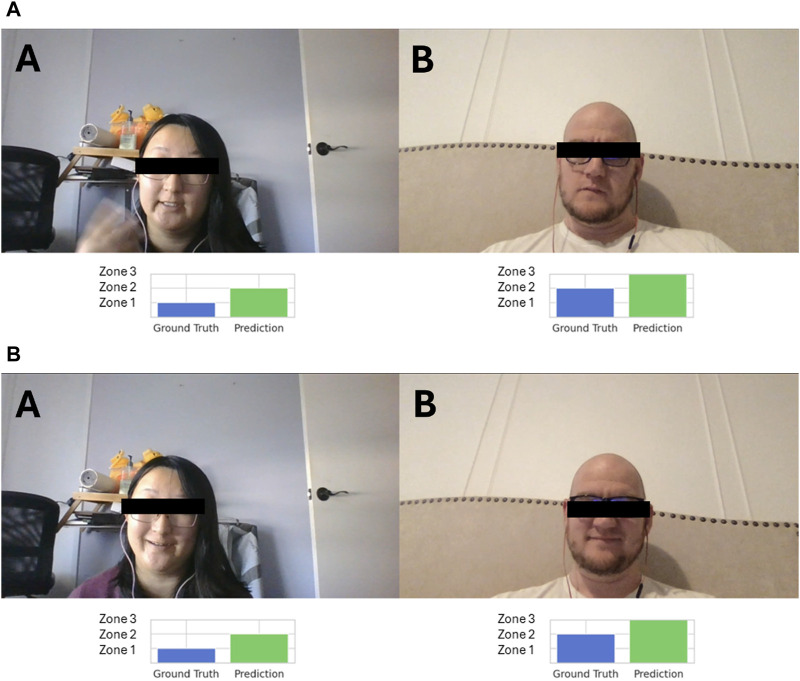
Two instances from a session between Participant A and Participant B where Participant A receives a low rating (*Zone 1)* from Participant B. This is a test session containing unseen data on which this model is evaluated. In both cases, the model fails to accurately classify *Zone 1* ratings. **(A)** An example of Participant B giving Participant A a low rating at 5:35. The model fails to predict this interpersonal rating accurately. **(B)** An example of Participant B giving Participant A a low rating at 8:43. Again, the model fails to predict this interpersonal rating accurately.

To understand why the model fails to correctly classify ratings from *Zone 1*, i.e., low interpersonal ratings, consider the following example. It examines instances from two distinct points in a conversation from the test data. Figures 11A, B both depict instances where Participant B gave a low rating to Participant A. It can be seen that in Figure 11A, Participant B has an apparent look of concern on their face while Figure 11B shows them smiling. On the other hand, Participant A’s facial expressions indicate engagement in both images. In both cases, Participant B was listening and Participant A was speaking, with no observable difference in their speaking style. Similarly, it is also evident that Participant B’s facial expressions are not consistent across the instances where they provide the same ratings to Participant A. This is not an isolated occurrence in this dataset. Given that *Zone 1* ratings are in minority in our training data (as illustrated in Figure 9), the chances of our network learning the behavioral patterns specific to this rating are reduced. The complexities of human interactions manifest in diverse combinations of audiovisual behavioral patterns that can indicate different interpersonal dynamics in different contexts. This calls for a representation of contextual factors and conversational content as part of the modeling approach.

**FIGURE 12:**
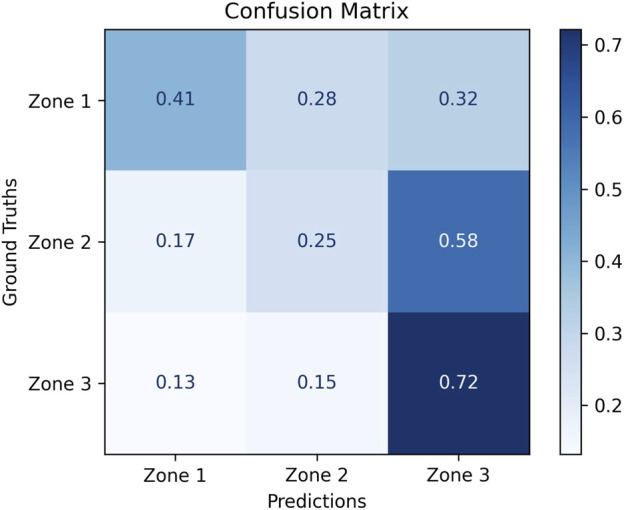
Class-normalized confusion matrix for our model that classifies relational affects into low (Zone 1), moderate (Zone 2), and high (Zone 3) affect zones.

**FIGURE 13:**
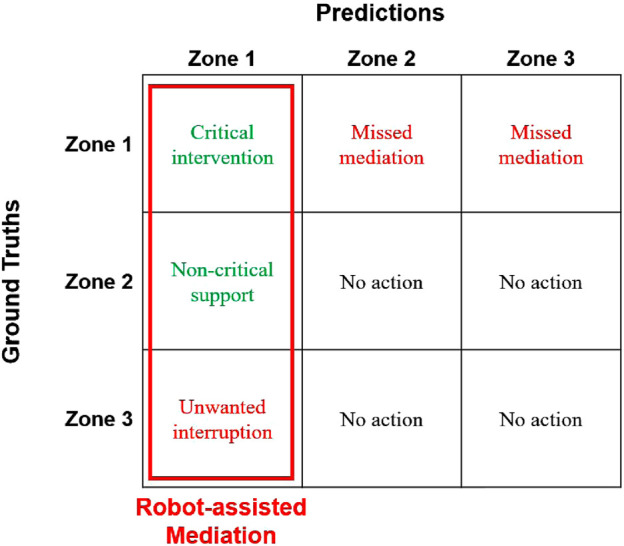
This figure can be compared with Figure 12 to evaluate how a robot using our model may perform as a mediator. The robot takes action when the model predicts Zone 1. When relational affect lies in Zone 1, and the model classifies it accurately as Zone 1, the robot is able to provide critical intervention. When Zone 1 is misclassified as Zone 2 or Zone 3, the robot misses opportunities to mediate an interaction. When Zone 2 and Zone 3 are misclassified as Zone 1, the robot’s actions are considered to provide non-critical support and unwanted interruptions respectively.

Next, we discuss the implications of our results for social mediation applications. To do so, we interpret our confusion matrix (Figure 12) from the perspective of impact on social mediation applications by comparing with the matrix in Figure 13. First, we establish that for a mediation application, it is critical that a robot takes action when relational affect is in Zone 1. In comparison, Zone 2 and Zone 3 indicate well-functioning interpersonal dynamics that do not necessitate active intervention. While the robot may still be able to provide some support by taking action to improve interpersonal dynamics in Zone 2, we view the absence of such supportive action to be a failure of mediation. This neutralizes the impact of the misclassifications of Zone 2 as Zone 3 (58%) shown in Figure 12. All misclassifications of Zone 1 as Zone 2 (28%) and Zone 3 (32%) indicate missed mediation opportunities and represent failure to provide necessary mediation. When Zone 1 is correctly classified as Zone 1, it indicates a need for critical intervention. A robot using our trained model is able to meet this expectation 41% of the time. When Zone 2 in incorrectly classified as Zone 1, it gives the robot an opportunity to offer non-critical support to improve the interpersonal dynamics. A robot trained on our model is able to offer non-critical support 17% of the time. When Zone 3 in incorrectly classified as Zone 1, it prompts the robot to take action when there is no need for intervention in a smoothly-functioning interaction. A robot trained on our model presents such unwanted interruptions 13% of the time. This analysis highlights the early capabilities of a social mediator robot: proactively intervening during negative interactions, enhancing neutral exchanges, all while respecting positive dialogues.

Human interactions are inherently intricate, involving subtleties and nuances that are made more complex due to individual dispositions, consequently adding to the challenge of developing generalizable computational models of relational affect. This is evident in our model’s performance since it does not account for the individual differences in our participants. The ratings in our dataset are highly subjective and intricately tied to individual differences in evaluating the interaction partners. Social psychology literature suggests that personality dispositions predict how individuals perceive, interpret, and react to social interactions with others. Studies have established that interpersonal evaluations do not only reflect the actual characteristics of the persons who are being rated, but also depend on the characteristics of those who provide the ratings (Kenny, 2004; Leising et al., 2015; Peabody, 1967; Saucier, 1994; Hannuschke et al., 2020). In particular, Neuroticism-related positivity bias in interpersonal perceptions has been found to cause raters to make more positive judgments of others’ sociability and warmth (Hannuschke et al., 2020). Neuroticism or negative affectivity is one of the personality traits in the Big Five model (McCrae and Costa, 1989), and is particularly suitable for investigating the influence of personality on interpersonal perceptions given its association with cognitive bias and selection tendencies (Finn et al., 2013; Marshall et al., 2015). Though personality analysis is beyond the scope of the present work, its connection to positivity bias may offer a possible explanation for the lack of negative ratings in our dataset. Additionally, individual differences may also dictate the usage style of a scale, such as extreme response style where raters tend to select responses at the extremes of the scales provided (Baird et al., 2017; Kenny et al., 2023). This may explain why +7 is the most frequently-selected rating in our dataset (see Figure 7). These characteristics of the dataset acknowledge that although self-reported, retrospective ratings are crucial for obtaining labels for interpersonal affective states from the raters’ own viewpoints, these can also be susceptible to biases that compromise their validity.

It is important to note that while this work provides insights into interpersonal dynamics within dyads, these dynamics may vary considerably in larger groups. For instance, even groups as small as three participants can form teams or sub-groups, where two individuals might align against the third, potentially creating in-group and out-group scenarios that directly impact interpersonal perceptions. Furthermore, the introduction of a robot as a mediator can significantly influence these dynamics. Previous research has demonstrated that a robot’s inclusion can affect human-human interactions in various ways (Weisswange et al., 2024), such as improving participation balance in conversations (Traeger et al., 2020), influencing conflict resolution strategies adopted by interactants (Shen et al., 2018), promoting inclusion (Gillet et al., 2020), and developing mutual understanding (Birmingham et al., 2020). This research demonstrates a robot’s ability to influence interpersonal interactions. However, questions regarding possible differences in behavioral cues exchanged between humans with and without the inclusion of a robot require further study.

A key factor in this work was the choice of the dataset and, hence, the corresponding task in which the interactions will be grounded. While this grounding is useful in eliciting goal-directed behavior—in this case, the negotiation between the participants—we were careful to choose a mainly conversational task that did not involve specialized movements or gestures for communication. This is unlike studies that utilizes tasks where physical movements may be the main indicators of participation within a team, e.g., assembly tasks (Haripriyan et al., 2024). Instead, we aimed to elicit natural conversational dynamics that may generalize to other conversational tasks as well. Having said that, our current model does not incorporate contextual factors, such as relationship, environmental setting, age, gender, and personality, among other factors, that may be crucial when evaluating the generalizability of a model to other conversational tasks. In addition to this, a negotiation inherently includes agreements, disagreements, persuasion, and collaborative decision-making, all of which can evoke diverse interpersonal dynamics. This is important for collecting a dataset containing samples of various interpersonal states from which machine learning models can learn. Thus, negotiation serves as a means to elicit these interpersonal dynamics, allowing us to model them using machine learning and apply the insights to other situations. The ratings estimated by our model can then be used by the robot mediator to devise an appropriate mediation strategy in line with the goals of the group and the target of mediation.

The goal of this work is to take steps towards the development of a computational model of relational affect that can be used to evaluate interpersonal dynamics in real-world social interactions in order to facilitate robot-assisted mediation applications. This goal guides our choice of dataset, our modeling techniques, and our evaluation of the model. While several models of group affect or other group behavior are available, we argue that their applicability for real-world deployment is limited due to the nature of the underlying training data, the constraints imposed by the modeling techniques on their generalization capabilities, or their application to very specific tasks. In contrast, our approach strives for real world applicability by 1) using a conversational dataset collected in the wild and is not task-specific; 2) capturing relational affect states rather than internal affect states that are more useful to evaluate interpersonal dynamics, 3) using a feature space that can be extracted in real time to enable real-world deployment in social mediation applications, and 4) employing a multi-perspective approach to model relational affect from the perspective of each individual interactant to enable the generation of effective and nuanced mediation strategies from the robot.

## 8 Limitations and future work

Currently, our model faces limitations in accurately classifying the three relational affect states, particularly the low states. To address this, incorporating individual differences among participants—such as personality traits or cultural backgrounds—could enhance the model’s ability to generalize learned affective representations. Additionally, we have yet to fully explore the untapped potential of speech content within the dataset. Analyzing speech patterns and linguistic cues may offer valuable insights into affective behaviors during dyadic interactions. Additional features that capture higher-level constructs, such as pauses, silences, gaze focus, and attention, may also impact the model’s ability to identify key interpersonal behavior. It is crucial to acknowledge that while the dataset is collected “in the wild,” the interactions occur in virtual settings. Consequently, we miss out on capturing additional behavioral cues like body pose and interpersonal distance, which are readily available in in-person interactions. These virtual dynamics may differ from the complexities encountered outside our dataset, which may have implications for mediation of in-person social interactions.

In future research, integrating personality traits and demographic information into the modeling process could help address the subjective nature of behavioral features and relational affect ratings. By considering individual differences in personality and demographics, the model may better capture the nuances of affective behaviors. Another promising avenue is leveraging pre-trained models. These models, often trained on large-scale datasets, have learned useful representations of language, vision, or other domains. Incorporating pre-trained embeddings or features into the existing model architecture could enhance its ability to learn from limited data.

Additionally, scaling to larger groups and real-world scenarios is crucial. Current work focuses on dyadic interactions but extending it to include larger groups can be valuable. In real-world scenarios (e.g., group discussions, meetings, or social gatherings), interpersonal dynamics involve complex interactions among multiple individuals. Capturing these dynamics requires modeling interpersonal behaviors taking place between all group members. Future work may explore how the existing approach can be adapted to handle group interactions by considering group-level features, temporal dependencies, and coordination patterns. Alternatively, other architectures may be utilized to capture the complexities of interactions in larger groups. For example, graph-based approaches are well-suited for capturing interpersonal relationships in larger groups, as they can effectively represent individual group members and their interactions, thereby capturing the complex dynamics within a group from each individual’s perspective.

Addressing these limitations and exploring the highlighted avenues will enhance the model’s practical utility and contribute to its value in robot-assisted social mediation applications. By enabling an understanding of the interpersonal dynamics in a human-human interaction, this work lays the groundwork for real-world applications of robot-assisted social mediation in group interactions.

## Data Availability

Publicly available datasets were analyzed in this study. This data can be found here: www.corae.org.

## Footnotes

1
https://https://corae.org/

2
https://imotions.com/products/imotions-lab/
