# Supplementary material for: Modeling interpersonal perception in dyadic interactions: towards robot-assisted social mediation in the real world
Source: Front Robot AI. 2024 Nov 28;11:1410957. doi: 10.3389/frobt.2024.1410957 (PMC11634758; doi:10.3389/frobt.2024.1410957)
Supplement: Supplementary file 1 [file DataSheet1.pdf]

Supplementary Material

1 SUPPLEMENTARY TABLES

Table S1. List of extracted audio features used to train our LSTM model.

#	Name	#	Name	#	Name	#	Name
1	ZCR	10	MFCC 2	19	MFCC 11	28	Chroma 7
2	Energy	11	MFCC 3	20	MFCC 12	29	Chroma 8
3	Energy Entropy	12	MFCC 4	21	MFCC 13	30	Chroma 9
4	Energy Centroid	13	MFCC 5	22	Chroma 1	31	Chroma 10
5	Energy Spread	14	MFCC 6	23	Chroma 2	32	Chroma 11
6	Energy Entropy	15	MFCC 7	24	Chroma 3	33	Chroma 12
7	Spectral flux	16	MFCC 8	25	Chroma 4	34	Chroma STD
8	Spectral Roll-off	17	MFCC 9	26	Chroma 5		
9	MFCC 1	18	MFCC 10	27	Chroma 6		

Table S2. List of extracted facial landmarks used to train our LSTM model.

#	Name	#	Name	#	Name
1	Right top jaw x	25	Nose tip x	49	Left lip corner x
2	Right top jaw y	26	Nose tip y	50	Left lip corner y
3	Right jaw angle x	27	Nose lower right boundary x	51	Left edge lower lip x
4	Right jaw angle y	28	Nose lower right boundary y	52	Left edge lower lip y
5	Gnathion x	29	Nose bottom boundary x	53	Lower lip center x
6	Gnathion y	30	Nose bottom boundary y	54	Lower lip center y
7	Left jaw angle x	31	Nose lower left boundary x	55	Right edge lower lip x
8	Left jaw angle y	32	Nose lower left boundary y	56	Right edge lower lip y
9	Left top jaw x	33	Outer right eye x	57	Bottom upper lip x
10	Left top jaw y	34	Outer right eye y	58	Bottom upper lip y
11	Outer right brow corner x	35	Inner right eye x	59	Top lower lip x
12	Outer right brow corner y	36	Inner right eye y	60	Top lower lip y
13	Right brow center x	37	Inner left eye x	61	Upper corner right eye x
14	Right brow center y	38	Inner left eye y	62	Upper corner right eye y
15	Inner right brow corner x	39	Outer left eye x	63	Lower corner right eye x
16	Inner right brow corner y	40	Outer left eye y	64	Lower corner right eye y
17	Inner left brow corner x	41	Right lip corner x	65	Upper corner left eye x
18	Inner left brow corner y	42	Right lip corner y	66	Upper corner left eye y
19	Left brow center x	43	Right apex upper lip x	67	Lower corner left eye x
20	Left brow center y	44	Right apex upper lip y	68	Lower corner left eye y
21	Outer left brow corner x	45	Upper lip center x		
22	Outer left brow corner y	46	Upper lip center y		
23	Nose root x	47	Left apex upper lip x		
24	Nose root y	48	Left apex upper lip y		
